# Preparation and Properties of Poly(ethylene glycol-co-cyclohexane-1,4-dimethanol terephthalate)/Polyglycolic Acid (PETG/PGA) Blends

**DOI:** 10.3390/polym13030452

**Published:** 2021-01-31

**Authors:** Kai Wang, Jianing Shen, Zhao Ma, Yipeng Zhang, Nai Xu, Sujuan Pang

**Affiliations:** 1 Department of Polymer Materials and Engineering, School of Materials Science and Engineering, Hainan University, Haikou 570228, China; wang_kai126@163.com (K.W.); shenjianing0823@163.com (J.S.); taohua20042@163.com (Z.M.); zhangyipeng2013@163.com (Y.Z.); 2 Department of Public Chemistry Teaching, School of Science, Hainan University, Haikou 570228, China; psjuan@hainanu.edu.cn

**Keywords:** PETG, PGA, tensile performance, interfacial compatibility, rheological property

## Abstract

Polyglycolic acid (PGA) is used as a reinforcing component to enhance the mechanical properties of poly(ethylene glycol-co-cyclohexane-1,4-dimethanol terephthalate) (PETG). The tensile performance, micromorphology, crystallinity, heat resistance, and melt mass flow rates (MFRs) of PETG/PGA blends with varying PGA contents were studied. Both the tensile yield strength and tensile modulus of the PETG/PGA blends increased gradually with an increase in the PGA content from 0 to 35 wt%. The tensile yield strength of the PETG/PGA (65/35) blend increased by 8.7% (44.38 to 48.24 MPa), and the tensile modulus increased by 40.2% (1076 to 1509 MPa). However, its tensile ductility decreased drastically, owing to the poor interfacial compatibility of PETG/PGA and the oversized PGA domains. A multiple epoxy chain extender (ADR) was introduced into the PETG/PGA (65/35) blend to improve its interfacial compatibility and rheological properties. The tensile performance, micromorphology, rheological properties, crystallinity, and heat resistance of PETG/PGA (65/35) blends with varying ADR contents were studied. The strong chain extension effect of ADR along with its reactive compatibilization improved the rheological properties and tensile ductility. By carefully controlling the ADR concentration, the performance of PETG/PGA blends can be regulated for different applications.

## 1. Introduction

Poly(ethylene glycol-co-cyclohexane-1,4-dimethanol terephthalate) (PETG) is an amorphous copolyester, synthesized by the polycondensation of 1,4-cyclohexanedimethanol (CHDM), ethylene glycol (EG), and terephthalic acid (TPA) in certain proportions [1].

PETG has excellent properties, such as high ductility, high chemical resistance, processability, and recyclability [2,3,4]. PETG, owing to its versatility, has been widely used for packaging. In recent years, PETG has also become a material of choice for 3D printing [5,6]. However, with its increased applications, the mechanical performance of PETG, such as tensile strength, modulus, and heat resistance, need to be boosted.

Seong-Hun et al. used carbon black (CB) as a reinforcement to enhance the mechanical properties of PETG [7]. The results revealed that when the CB content was increased from 0 to 3 wt%, the tensile yield strength and tensile modulus of the composite increased by 11.2 and 5.9%, respectively. This could be attributed to the excellent mechanical properties and three-dimensional crystal structures of CB particles, which increased the strength of the PETG.

Yuhsin Tsai et al. studied the influence of the organic clay content on the physical properties of PETG [8]. It was found that with an increase in the organic clay content from 0 to 3 wt%, the tensile yield strength increased from 47.9 to 53.3 MPa, and the tensile modulus increased from 1849 to 2154 MPa. This was mainly due to the reinforcing effect of the clay nanosheets, which were rigid and had a high aspect ratio. However, the tensile ductility (in terms of the elongation at break) of the PETG/organoclay nanocomposites significantly declined with an increase in organic clay content. For example, when 3 wt% organic clay was introduced into the PETG matrix, the elongation at break decreased from 112.9% (neat PETG sample) to 9.2%. As well-known, for most polymer blends and composites, how to tailor their properties to achieve a stiffness–ductility balance is still an interesting and challenging issue. 

Besides rigid inorganic particles, rigid organic polymers are also potential reinforcements for the PETG matrix. To date, few studies have reported on the introduction of rigid polymers into the PETG matrix as the reinforcing material.

Polyglycolic acid (PGA) is a kind of aliphatic polyester, mainly synthesized by the ring-opening polymerization of glycolide or by the direct polycondensation of glycolic acid [9,10]. PGA possesses excellent mechanical strength and rigidity [11,12,13] along with good biocompatibility and biodegradability [14,15]. Owing to its high strength and rigidity (tensile strength = 115 MPa and tensile modulus = 7 GPa) [11,12], PGA can be considered as a candidate for enhancing the mechanical performance of PETG.

To date, very little research has been reported on the reinforcing effect of PGA on PETG. In this paper, PGA was used as a reinforcing component to improve the mechanical properties of PETG. PETG/PGA binary blends with different PGA contents were prepared by melt-blending. The tensile performance, micromorphology, crystallinity, heat resistance, and melt mass flow rates (MFRs) of the PETG/PGA blends were studied in detail. It was found that the tensile yield strength and modulus of the binary blend increased with an increase in the PGA content. With 35 wt% PGA, the PETG/PGA (65/35) blend showed the maximum tensile yield strength and modulus. However, the poor interfacial compatibility between the PETG matrix and the PGA domains caused the tensile ductility of the PETG/PGA (65/35) blend to drastically decrease. Moreover, the high melt fluidity of PGA increased the MFR of the PETG/PGA blend, which was not conducive to its processing for some common processing technologies, such as extrusion, blown film, thermoforming, etc. The interfacial compatibility and the rheological properties of the PETG/PGA (65/35) blend could be improved by introducing a multiple epoxy chain extender into the PETG/PGA (65/35) blend. The effect of the chain extender on the PETG/PGA melt was evaluated by torque rheometry and MFR testing. In addition, the effect of adding the chain extender on the tensile properties, micromorphology, crystallinity, and heat resistance of the PETG/PGA (65/35) blend was studied in detail. Finally, a modified PETG/PGA (65/35) blend with improved tensile performance and heat resistance, and good rheological properties was successfully prepared.

## 2. Materials and Methods

### 2.1. Materials 

**Figure 1:**
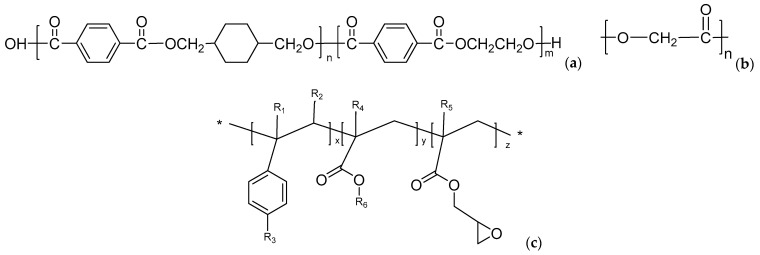
Chemical structures of (**a**) poly(ethylene glycol-co-cyclohexane-1,4-dimethanol terephthalate) (PETG), (**b**) polyglycolic acid (PGA), and (**c**) Joncryl ADR 4370s (ADR).

PETG (K2012), with a density of 1.27 g/cm^3^ and MFR of 5.1 g/10 min (230 °C, 2.16 kg), was supplied by SK Chemicals Co. Ltd. (Seoul, Korea). PGA, with a density of 1.53 g/cm^3^ and MFR of 28.4 g/10 min (230 °C, 2.16 kg), was obtained from Inner Mongolia Pujing Polymer Materials Technology Co. Ltd. (Bao Tou, China). The multiple epoxy chain extender (Joncryl ADR 4370s, abbreviated as ADR in this paper) was purchased from BASF (Ludwigshafen, Germany). Its molecular weight and epoxy equivalent weight were 6800 and 285 g/mol, respectively. The chemical structures of PETG, PGA, and ADR are shown in Figure 1.

### 2.2. Sample Preparation 

Prior to compounding, pellets of both PETG and PGA were dried under vacuum at 45 °C for 12 hours to remove moisture. An internal mixer (XS-60, Shanghai Kechuang Rubber and Plastic Machinery Equipment Co. Ltd., Shang Hai, China) equipped with a 60 mL mixing chamber was used for (i) the melt-blending of PETG/PGA mixtures with different PGA contents, and (ii) the reactive melt-blending of PETG/PGA/ADR (65/35/x) blends with different ADR contents. The compositions of the PETG/PGA (y/z) and PETG/PGA/ADR (65/35/x) samples are presented in Table 1, where y/z and 65/35 denote the weight ratio of the PETG/PGA, and x denotes the ADR content in parts per hundred of total resin (phr). The melt blending was conducted at 230 °C for 8 min at a rotational speed of 50 rpm. Then, the obtained blends were compression-molded using a hot press at 230 °C under 20 MPa for 8 min. After hot pressing, the closed mold with the in-mold melt was quickly transferred into a water-cooling system with a clamping pressure of 20 MPa and cooled down to room temperature at about 100 °C/min. Thus, compression-molded sheets of 0.5 mm thickness were obtained. These sheets were cut into dumbbell-shaped specimens for the subsequent tensile test.

### 2.3. Tensile Testing

A universal tensile testing machine (WDW-1, Yinuo Century Testing Instrument Co. Ltd., Ji Nan, China) was used to determine the tensile performance of the samples. Prior to tensile testing, the samples were stored in an electronic drying oven for 48 h. The tensile yield strengths, tensile moduli, and elongation at break values of the samples were measured at a tensile rate of 5 mm/min. Each result presented in Table 2 is an average of five valid test results per sample, along with the standard deviation.

### 2.4. Scanning Electron Microscopy (SEM)

SEM (Verios G4 UC, Thermo Fisher Scientific Brno Co. Ltd., Waltham, USA) was used to study the morphological evolutions of the specimens during the tensile deformation process. The tensile fractured specimens were collected after the tensile testing. Some tensile fractured specimens were immersed in liquid nitrogen for a sufficient time, and then broken quickly to obtain cryofractured surfaces along the tensile direction. The cryofractured surfaces of the specimens were sputter coated with gold powder in order to obtain better SEM images. Both the stretched and unstretched regions on the cryofractured surface of each specimen were photographed. 

### 2.5. Differential Scanning Calorimetry (DSC)

DSC curves were obtained using a DSC instrument (Q100, TA Instruments, New Castle, DE, USA). The DSC thermograms were used to determine the crystallinities of the samples. The specific steps were as follows: each sample (around 5–8 mg) was heated from 20 to 250 °C at 10 °C/min in an N_2_ atmosphere to obtain the heating DSC thermogram. The relevant parameters were determined from the DSC thermogram, and the crystallinity (*X*_c_) of the PGA component was calculated according to Equation (1).

(1) $$X_{c}=\frac{\left|\Delta H_{m}\right|}{\omega_{PGA}\times \left|\Delta H_{m}^{0}\right|}\times 100{\%}_{\;}$$

where ω*_PGA_* is the mass fraction of the PGA component in the sample, Δ*H*_m_ is the endothermic melting enthalpy of the PGA component, and $\Delta H_{m}^{0}$ is the ideal melting enthalpy of 100% crystalline PGA (−198.14 J/g) [16,17]. In this study, the endothermic enthalpy was negative in the DSC measurements.

### 2.6. Wide-Angle X-ray Diffraction (WAXD)

The crystalline structures of the samples were determined by WAXD (Smart Lab, Rigaku Co., Akishima, Japan). The dimensions of the specimens were 40 × 40 × 2 mm (length × width × thickness). A CuKα radiation source (λ = 1.542Å) was used at a 40 kV voltage and 40 mA current. WAXD data were recorded in the *2θ* range of 5−40° with a scanning speed of 5°/min.

### 2.7. Vicat Softening Temperature (VST)

A VST tester (CZ-6005, Changzhe Testing Machinery Co., Ltd., Yang Zhou, China) was used to determine the heat resistance of the samples. The VST test was conducted according to GB/T 1633–2000 at a heating rate of 120 °C/h and under a load of 10 N. The dimensions of the specimens were 10 × 10 × 2 mm (length × width × thickness). 

### 2.8. Torque

The curves for the torque vs. mixing time of the PETG/PGA/ADR (65/35/x) blends were plotted from the results obtained by torque rheometry (XSS-300, Kechuang Rubber & Plastic Equipment Co. Ltd., Shang Hai, China). A steady temperature of 230.0 ± 1.0 °C was maintained, and the rotation speed was set at 50 rpm. The total mixing time was set as 30 min.

### 2.9. Melt Mass Flow Rate (MFR)

The MFRs of the samples were determined using a melt flow rate apparatus (XNR-400B, Desheng Testing Equipment Co. Ltd., Cheng De, China) at 230 °C with a 2.16 kg load.

### 2.10. Transmission Electron Microscopy (TEM)

Transmission electron microscopy (JEM-2100, JEOL, Akishima, Japan) was employed to investigate the influence of adding ADR on the domain size of PGA at an accelerating voltage of 200 kV. TEM images of microtomed sections of 70–80 nm thickness were obtained.

## 3. Results and Discussion

### 3.1. PETG/PGA Binary Blends 

#### 3.1.1. Tensile Performance

**Figure 2:**
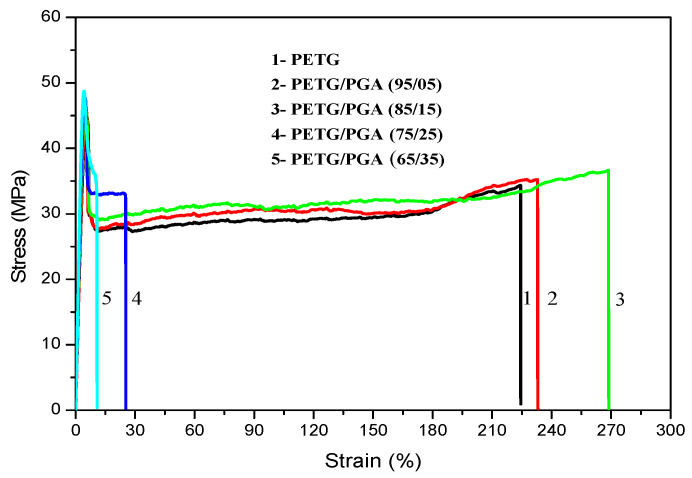
Tensile strain–stress curves of PETG/PGA samples.

The tensile yield strengths, tensile moduli, and elongation at break values of PETG/PGA binary samples with various PGA contents are listed in Table 2. The typical stress–strain curves are shown in Figure 2. 

As shown in Table 2, with an increase in PGA content from 0 to 35 wt%, the tensile yield strength of the PETG/PGA binary sample increased from 44.38 MPa (neat PETG) to 48.24 MPa. Meanwhile, the tensile modulus increased from 1076 to 1509 MPa, indicating that PGA has a good reinforcing effect on the PETG matrix. However, when the PGA content was more than 15 wt%, the tensile ductility (in terms of the elongation at break) of the binary sample decreased remarkably. With an increase in PGA content to 35 wt%, the elongation at break decreased from 223.0% (neat PETG) to 11.1%. This could be attributed to the poor interfacial compatibility between PETG and PGA. With an increase in PGA content, a serious combination of the PGA dispersed phase was inevitable as a result of the poor interfacial compatibility. The oversized PGA particles with poor interfacial adhesion caused defects in the PETG matrix that induced the formation of cracks, which developed rapidly under tensile stress. Similar experimental results have also been reported for many other blends [18,19,20].

#### 3.1.2. Scanning Electron Microscopy (SEM)

**Figure 3:**
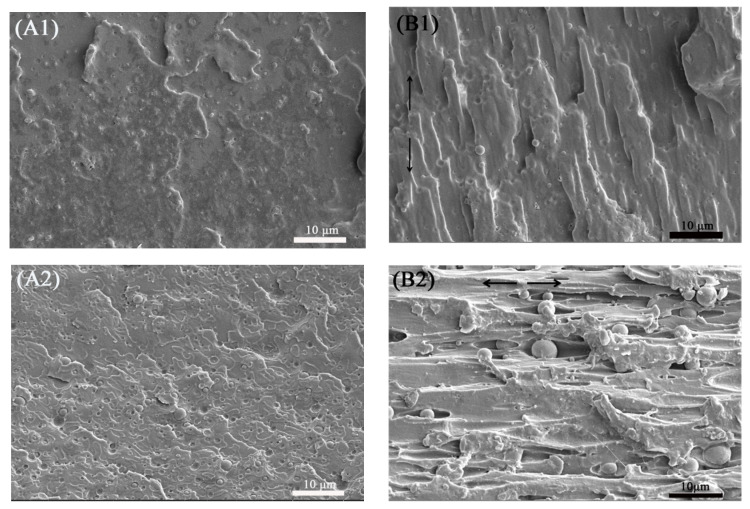

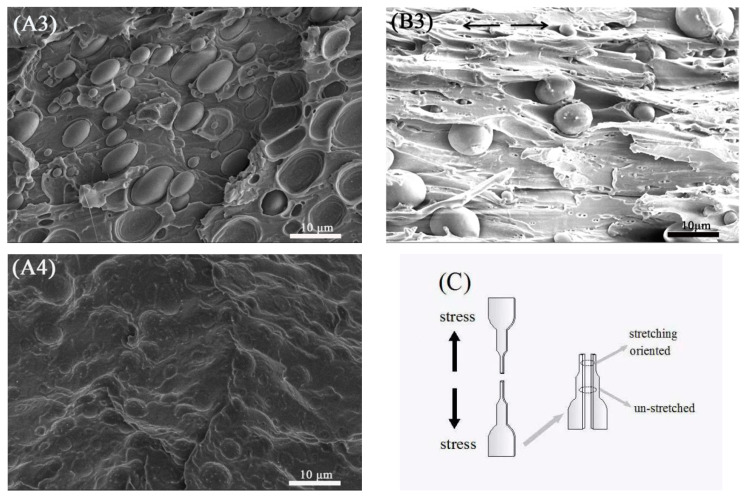
SEM images (×3000) of cryofractured surfaces of PETG/PGA tensile fractured specimens along tensile direction. (**A**) unstretched part ((**A1**)-95/5, (**A2**)-85/15, (**A3**)-75/25, (**A4**)-65/35), (**B**) stretching-oriented part ((**B1**)-95/5, (**B2**)-85/15, (**B3**)-75/25), and (**C**) sketching diagram of cryofractured surfaces along tensile direction.

To study the micromorphological evolution of the binary samples during the tensile process, the cryofractured surfaces of the tensile fractured specimens along the tensile direction, including the stretching-oriented parts and the unstretched parts, are shown in Figure 3. 

The unstretched regions of the PETG/PGA (95/5) and (85/15) samples (see Figure 3(A1,A2)) showed that the PGA particles with finer particle sizes (<5 μm for most of the PGA particles) uniformly dispersed in the PETG matrix. However, as the PGA content increased to 25 and 35 wt%, a serious combination of PGA domains appeared, as shown in Figure 3(A3,A4). Most of the PGA particles in the PETG/PGA (75/25) and (65/35) samples showed oversized diameters (more than 5 μm). The formation of such oversized PGA particles could be attributed to the poor interfacial compatibility in the PETG/PGA blend and increased PGA content. 

For the PETG/PGA (95/5) and (85/15) samples (see Figure 3(B1,B2)), the regions oriented in the stretching direction showed the debonding of PGA particles from the PETG matrix along with highly oriented cavities. The tensile performance shown in Table 2 confirmed that the PGA component had an obvious reinforcing effect on the PETG matrix. In addition, when the PETG/PGA (95/5) and (85/15) specimens were subjected to tensile loads, finer PGA particles showed stronger stress concentration around these particles, which led to the formation of massive shear zones in the PETG matrix. However, no plastic deformation occurred in the PGA domains, due to their superior rigidity. As a result, interfacial debonding in PETG/PGA was observed in the SEM images. The cavities formed due to interfacial debonding deformed along the tensile direction. This was followed by the plastic deformation and stretching of the PETG matrix along the tensile direction, as shown in Figure 3(B1,B2). Compared to that of the neat PETG sample, the presence of fine particles of PGA induced a higher number of shear zones in the matrix under a tensile load. Therefore, the improvement in the fracture toughness was due to energy absorption, caused by the matrix deformation. This was the result of the centralization of external stress around the PGA particles. Furthermore, these shear zones could effectively terminate crazes and prevent crack propagation. Consequently, the tensile yield strength, modulus, and elongation at break of the PETG/PGA (85/15) sample were improved. With an increase in the PGA content to 25 wt%, many oversized PGA particles dispersed in the PETG matrix, as shown as Figure 3(B3). As we know, dispersed particles with excessive domain sizes have relatively less ability to induce the formation of shear zones in the matrix that weaken the craze termination ability of the matrix. Meanwhile, the oversized PGA particles with poor interfacial compatibility became defect points and induced the formation of massive cracks under a tensile load. This led to the formation of an unstable and premature fracture. As a result, the PETG/PGA (75/25) sample showed a considerable decrease in its tensile ductility. A further increase in PGA content to 35 wt% led to the formation of more oversized PGA particles in the PETG matrix. This led to a further decrease in the tensile ductility of the binary sample that even caused a ductile–brittle transition in the tensile behavior. Owing to its poor tensile ductility, the PETG/PGA (65/35) sample (with a very low elongation at break of 11.1%) showed no obvious tensile yield and orientation behavior before its rupture during the tensile testing. Hence, an SEM image of the stretching-oriented part for the PETG/PGA (65/35) sample cannot be provided in this paper.

#### 3.1.3. DSC and WAXD Measurements

It is well-known that the crystallization properties of thermoplastic polymers, such as the crystallinity, crystalline structures, etc., can significantly influence the physical performance of the polymers and their blends [21,22,23,24]. 

**Figure 4:**
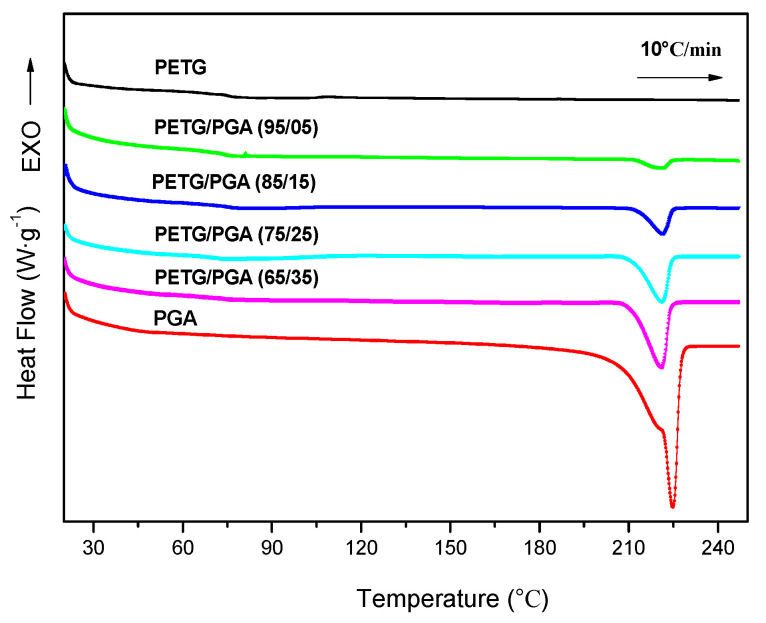
Heating DSC thermographs of PETG/PGA (y/z) samples.

DSC and WAXD analysis were used to evaluate the crystallization properties of PETG/PGA blends with different PGA contents. The heating DSC thermograms of neat PETG, PGA, and PETG/PGA binary samples are shown in Figure 4. The corresponding thermodynamic parameters are summarized in Table 3. 

**Figure 5:**
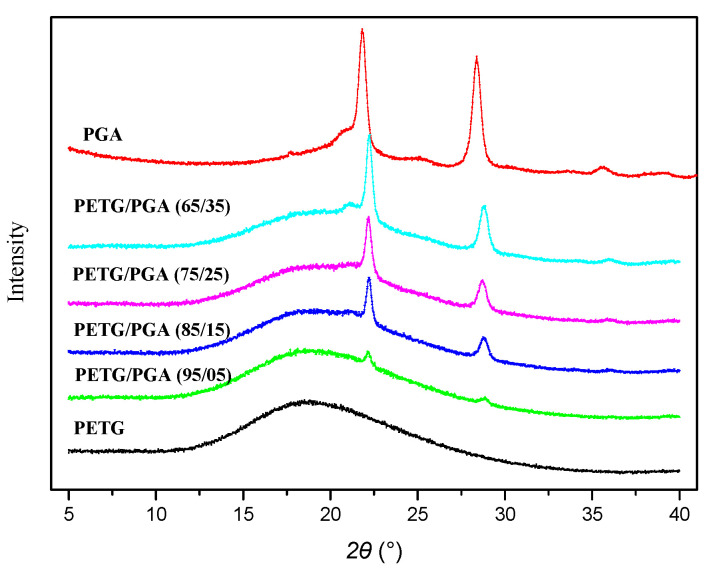
Wide-angle X-ray diffraction (WAXD) patterns of PETG, PGA, and PETG/PGA (y/z) samples.

The DSC thermogram of the neat PETG sample displayed a heat jump to approximately 74.9 °C, which corresponded to its glass transition temperature (*T*_g_). Moreover, the absence of an endothermic peak of melting proved that PETG was an amorphous polyester without crystallization ability. A similar conclusion was also drawn from its WAXD analysis (see Figure 5). 

On the other hand, the DSC thermogram of the neat PGA sample showed its melting temperature (*T*_m_) to be approximately 224.9 °C, along with a sharp endothermic melting peak. The DSC thermograms of the binary PETG/PGA samples showed no obvious shifting of peaks for the *T*g of the PETG matrix and *T*_m_ of the PGA component. Moreover, in contrast to the amorphous nature of the neat PETG sample, the neat PGA sample showed a semicrystalline structure with a relatively high crystallinity of 37.7%. For the PETG/PGA binary samples with lower PGA contents (≤15 wt%), the crystallinity of the PGA component was decreased compared to that of the neat PGA, as seen in Table 3. The reason for this is as follows: with a lower PGA content, some of the PETG and PGA macromolecular chains or segments could interpenetrate each other. As a result, the crystallization ability of the PGA component was restrained, which led to a decrease in the crystallinity of the PGA component. However, with an increase in the PGA content (≥25 wt%), a serious combination of the PGA domains caused by the microphase separation in the binary blending system occurred, which weakened the interactions between the PETG and PGA chains and segments. Hence, the crystallinity of the PGA increased with an increase in the PGA content. Compared to the crystallinity (37.7%) of the neat PGA sample, the crystallinity of the PGA component in the PETG/PGA (65/35) sample was restored to 36.0%.

The WAXD patterns of the PETG/PGA binary samples with various PGA contents are shown in Figure 5. For comparison, the WAXD patterns of the neat PETG and neat PGA are also provided. Consistent with the DSC results, the WAXD pattern of the neat PETG showed a dispersive diffraction peak in the range of *2θ* = 10° to 35°, indicating that the neat PETG was amorphous. Neat PGA showed two sharp diffraction peaks at 2θ = 21.8° and 28.4°, corresponding to the (110) and (020) planes of PGA crystals, respectively [25,26,27]. In the WAXD pattern of PETG/PGA (95/5), two sharp diffraction peaks at 22.2° and 28.9° were characteristic of PGA crystals. Moreover, the dispersive diffraction peak from 2θ = 10° to 35° indicated that the PETG matrix was completely amorphous. With an increase in PGA content, the two diffraction peaks characteristic of PGA crystals in the binary samples increased gradually, as expected.

#### 3.1.4. Vicat Softening Temperature (VST)

The results of the DSC and WAXD analyses confirmed that the PETG matrix was amorphous, regardless of the PGA content. By contrast, the PGA component was semicrystalline, with higher crystallinity. Hence, theoretically, the presence of PGA domains could improve the heat resistance of PETG. 

**Figure 6:**
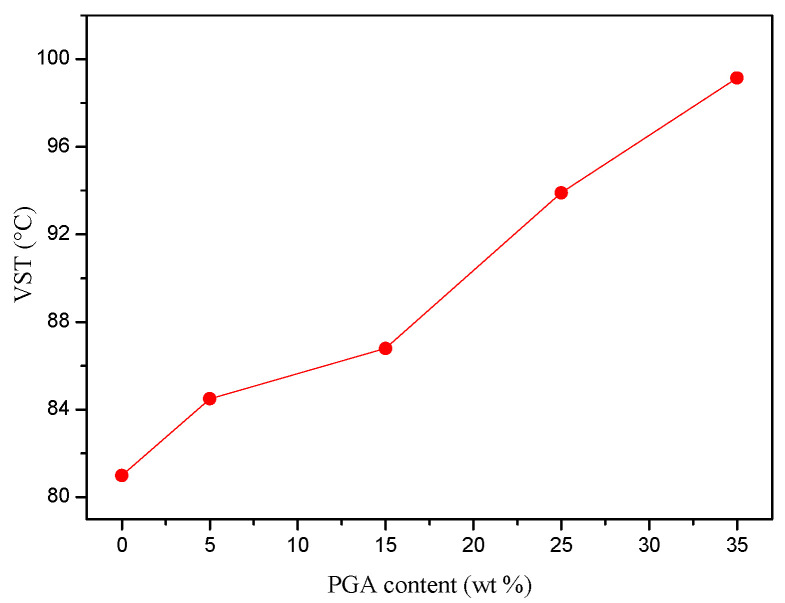
Influence of PGA content on Vicat softening temperature (VST) of PETG/PGA samples.

The VST is one of the most important parameters for evaluating the heat resistance of a thermoplastic polymer. For an amorphous thermoplastic resin, the VST is strongly affected by its *T*_g_. Due to its low *T*_g_ (ca. 75.0 °C), neat PETG showed poor heat resistance, which limits its applications at high temperatures. The relationship between the VST and PGA content is shown in Figure 6. As expected, the heat resistance of the PETG/PGA blends increased with an increase in PGA content. When the PGA content increased from 0 to 35 wt%, the VST increased from 81.0 °C (neat PETG) to 99.2 °C. This could be attributed to the reinforcement of the PGA particles on the amorphous PETG matrix, owing to the rigidity and semicrystalline nature of PGA.

#### 3.1.5. Melt Mass Flow Rate (MFR)

**Figure 7:**
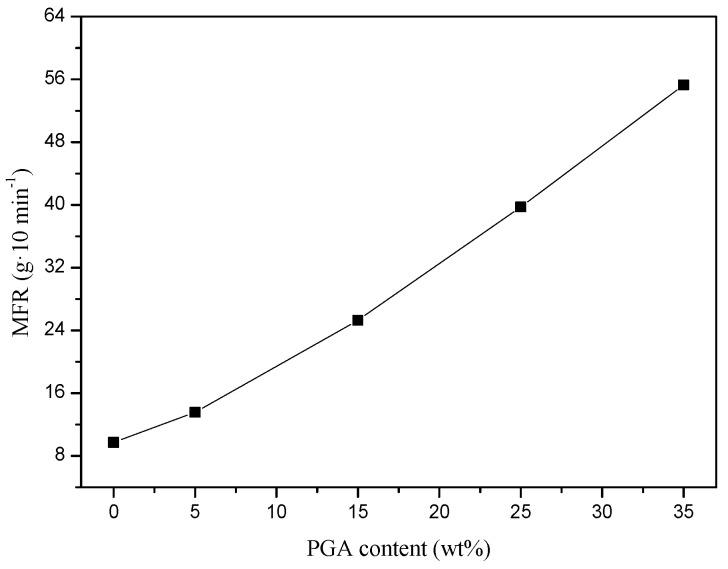
Influence of PGA content on MFR of PETG/PGA samples.

The MFR values of binary blends with varying PGA contents are shown in Figure 7. The MFR increased with an increase in PGA content, due to the high melt fluidity of PGA. When the PGA content increased from 0 to 35 wt%, the MFR value (230 °C, 2.16 kg) increased rapidly from 9.7 to 55.3 g/10 min. It should be noted that the PETG/PGA blends with high melt fluidity were not suitable for some of the important processing methods, such as extrusion, blown film, thermoforming, etc. 

With the incorporation of 35 wt% PGA into the PETG matrix, the binary sample showed the maximum tensile yield strength and modulus. However, the PETG/PGA (65/35) blend displayed very low tensile ductility, which could be attributed to the oversized PGA particles and poor interfacial compatibility in PETG/PGA. Moreover, the addition of PGA with high melt fluidity resulted in an excessively high MFR for the PETG/PGA (65/35) blend that significantly weakened its processing stability. Hence, a multiple epoxy chain extender (ADR) was used to increase the melt viscosity of the PETG/PGA (65/35) blend through a chain extension/branching reaction between the polyester chains and ADR. This facilitated further processing, since a high melt viscosity is a prerequisite for processes such as extrusion, blown film, thermoforming, etc. Moreover, ADR was also expected to act as a reactive compatibilizer to improve the interfacial compatibility of PETG/PGA, decrease the PGA domain size, and thus improve the tensile ductility of the PETG/PGA (65/35) blend.

### 3.2. PETG/PGA/ADR Ternary Blend

#### 3.2.1. Torque and MFR

**Figure 8:**
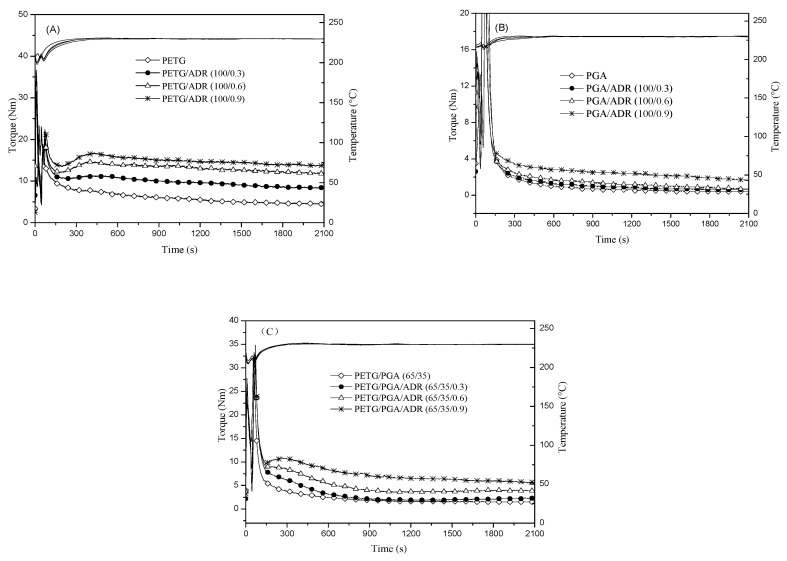
Torque vs. time curves for (**A**) PETG/ADR (100/x), (**B**) PGA/ADR (100/x), and (**C**) PETG/PGA/ADR (65/35/x) (x = 0, 0.3, 0.6, and 0.9).

Rheological tests of neat PETG, neat PGA, and PETG/PGA (65/35) blends with and without ADR were carried out by torque rheometry, and the results are shown in Figure 8. Experiments were conducted to study the chain extension reaction of the polyesters as a function of the mixing time in the molten state. Figure 8(A,B) show that the torque curves of neat PETG and neat PGA gradually shifted upward with an increase in ADR content. According to previous research [20,28,29,30], the multifunctional epoxide groups of the chain extender could react with both the carboxyl and hydroxyl end groups of many kinds of polyester. The resultant chain extension/branching reactions are responsible for the increase in melt viscosity of polyesters during melt processing. Theoretically, the multifunctional epoxide groups of ADR could react with both the carboxyl and hydroxyl end groups of PETG or PGA chains. This led to chain extension/branching reactions between the polyesters and ADR. Hence, the increase in viscosity could be explained by the formation of chain extended/long-branched PETG or PGA chains with ADR modification. 

The torque value of the PETG/PGA (65/35) blend increased upon the addition of ADR, as shown in Figure 8C. However, the chain extension/branching reactions of the PETG/PGA/ADR (65/35/x) blends were more complicated. In addition to PETG–ADR–PETG and PGA–ADR–PGA with chain extension/long-branched structures, the PETG–ADR–PGA copolymer could also be generated in situ through chain extension/branching reactions during blending.

**Figure 9:**
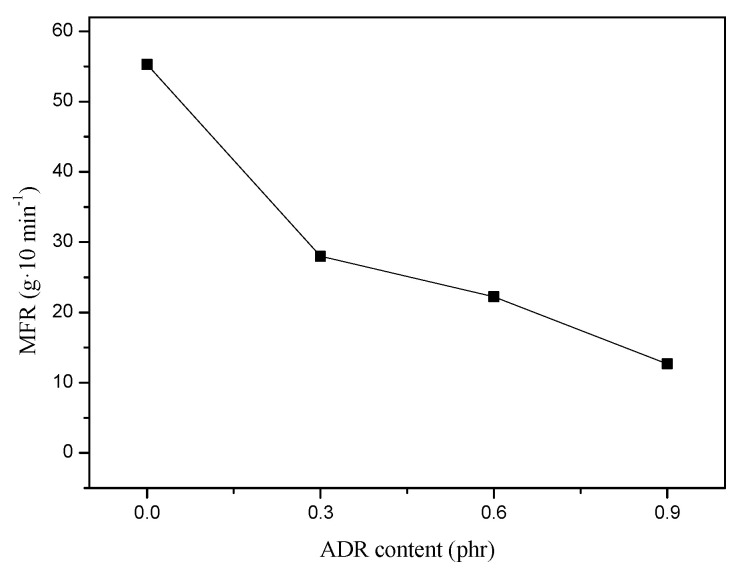
Effect of ADR addition on MFR of PETG/PGA/ADR (65/35/x) samples.

MFR testing was conducted to further evaluate the impact of the ADR concentration on the melt fluidity of the PETG/PGA/ADR (65/35/x) samples. A plot of MFR versus ADR content is shown in Figure 9. The addition of ADR resulted in a pronounced decrease in the MFR of the PETG/PGA/ADR blends. The introduction of 0.9 phr of ADR into the blend led to a decrease in the MFR value from 55.3 g/10 min for the PETG/PGA (65/35) binary sample to 12.7 g/10 min. This could be mainly attributed to the chain extension/branching reactions between the polyesters and ADR. Based on the rheological characteristics during polymer processing and studies on PLA/PBAT blends [20], it could be confirmed that the increase in molecular weight and the formation of long-branched structures were key factors that increased the melt viscosity of the polyesters. These were a result of modification with multiple epoxy chain extenders.

Obviously, an increase in the viscosity of a PETG/PGA/ADR (65/35/x) sample could facilitate its further processing, since a high melt viscosity is a prerequisite for processes such as extrusion, blown film, thermoforming, etc. Moreover, since the multiple epoxy groups of ADR could react with the terminal –OH and –COOH groups of PETG and PGA, theoretically, ADR could be used as a reactive compatibilizer to improve the interfacial compatibility in PETG/PGA blends. In this manner, the tensile performance and micromorphology of the PETG/PGA blend could be regulated by controlling the ADR content. 

#### 3.2.2. Tensile Performance

**Figure 10:**
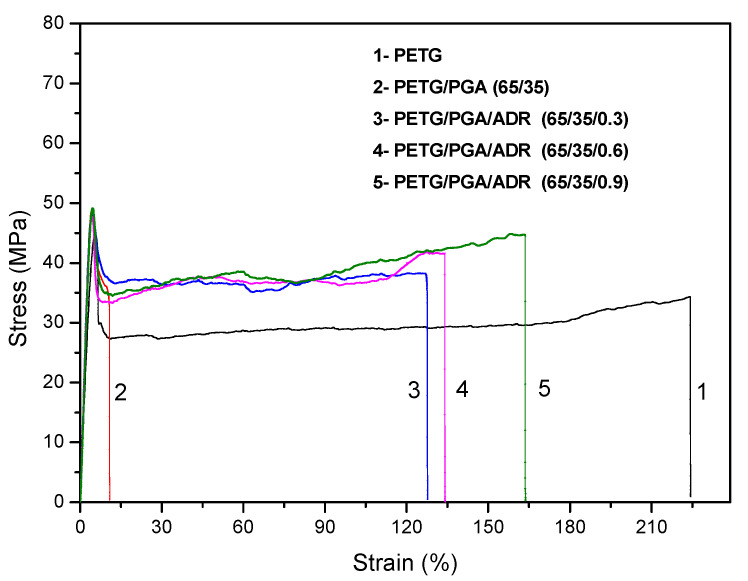
Tensile stress–strain curves for neat PETG and PETG/PGA/ADR (65/35/x) samples.

The tensile properties of the neat PETG and PETG/PGA/ADR (65/35/x) blends are listed in Table 4. The typical tensile stress–strain curves of these samples are shown in Figure 10. Compared to neat PETG, the PETG/PGA (65/35) blend showed poor tensile ductility, due to a drastic decrease in the elongation at break from 223.0% (neat PETG) to 11.1%. It is worth noting that the elongation at break of the PETG/PGA/ADR (65/35/x) samples increased with an increase in the ADR content. Upon the addition of 0.3 phr of ADR, the elongation at break increased from 11.1% for the binary sample to 123.2%, an increase of 1010%. With the further addition of 0.9 phr ADR, the elongation at break of the ternary sample reached 160.8%. This improvement in tensile ductility could be attributed to the reactive compatibilization effect of ADR on the PETG/PGA interface. On the other hand, both the tensile yield strength and the tensile modulus of the PETG/PGA (65/35) binary sample were markedly higher than those for neat PETG, due to the reinforcing effect of PGA. Furthermore, compared to the PETG/PGA (65/35) binary sample, the tensile yield strengths of the PETG/PGA/ADR (65/35/x) samples remained unchanged, with only a slight decrease in tensile modulus with an increase in ADR content from 0 to 0.9 phr. From Table 4, it can be observed that the PETG/PGA (65/35) blend modified with 0.9 phr of ADR showed the most balanced tensile performance. Compared to neat PETG, the PETG/PGA/ADR (65/35/0.9) sample showed an enhanced tensile yield strength and tensile modulus, which corresponded to increases of 9.1 and 30.4%, respectively. At the same time, the ternary sample also showed relatively good tensile ductility, with an elongation at break of 160.8%. 

In summary, with ADR chain extension and compatibilization, the rheological properties and tensile ductility of the PETG/PGA/ADR (65/35/x) blend were greatly improved.

#### 3.2.3. TEM Observation

Microstructural evolution is known to greatly influence the mechanical performance of a blend. Hence, TEM analysis was used to investigate the impact of adding ADR on the micromorphology of PETG/PGA/ADR (65/35/x) samples with varying ADR contents. This would help to further elucidate the relationship between the tensile performance and micromorphology of the samples with different ADR contents. 

**Figure 11:**
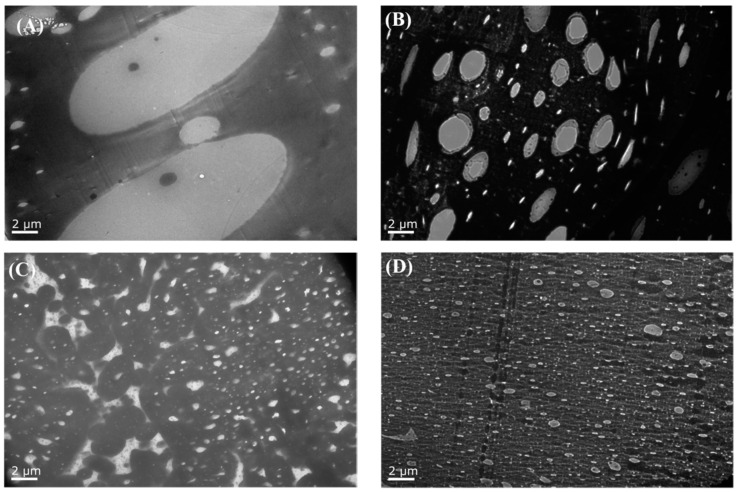
TEM images (×1000) of PETG/PGA/ADR (65/35/x; x = 0 (**A**), 0.3 (**B**), 0.6 (**C**), and 0.9 (**D**)) samples.

**Figure 12:**
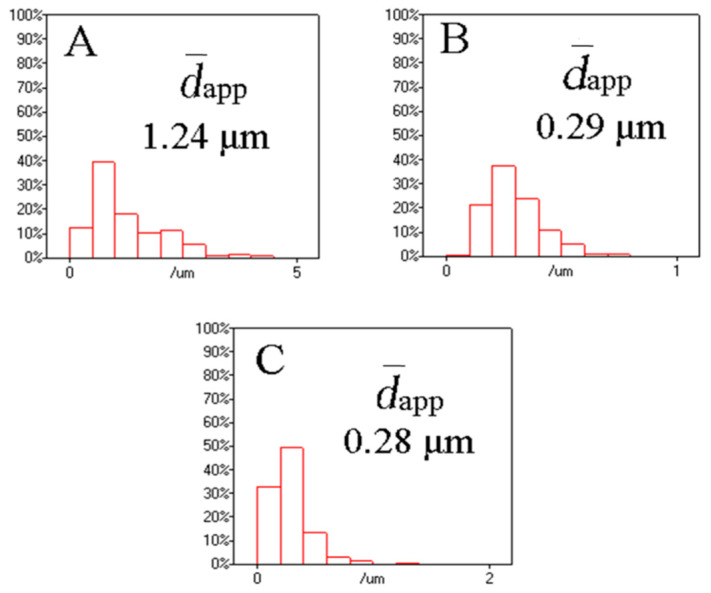
Statistical results for apparent particle sizes of PETG/PGA/ADR (65/35/x; x = 0.3 (**A**), 0.6 (**B**), and 0.9 (**C**)) samples.

The typical TEM images of the PETG/PGA/ADR (65/35/x) samples at 1000x magnification are presented in Figure 11. The range of the particle size distribution and apparent number-average particle sizes (${\overset{-}{d}}_{\mathrm{app}}$) of PGA for the ternary samples were obtained using average diameter analysis software (Nano Measurer 1.2), as shown in Figure 12. 

TEM analysis showed the presence of many oversized PGA particles dispersed in the PETG/PGA (65/35) binary sample. This was due to the serious combining of the PGA domains as a result of the poor interfacial compatibility in the PETG/PGA blending system. However, upon the addition of 0.3 phr of ADR, the ${\overset{-}{d}}_{\mathrm{app}}$ of the PGA domains decreased significantly. When the amount of ADR increased from 0.3 to 0.6 phr, the ${\overset{-}{d}}_{\mathrm{app}}$ further decreased from 1.24 to 0.29 μm. As the amount of added ADR increased to 0.9 phr, the value of ${\overset{-}{d}}_{\mathrm{app}}$ no longer decreased. That could be explained by the fact that the chain extension/branching reactions of PETG, PGA, and ADR could form some PETG–ADR–PGA copolymers in situ. These copolymers could improve the PETG/PGA interfacial adhesion and thus significantly reduce the size of the PGA domains. 

#### 3.2.4. SEM Observation

**Figure 13:**
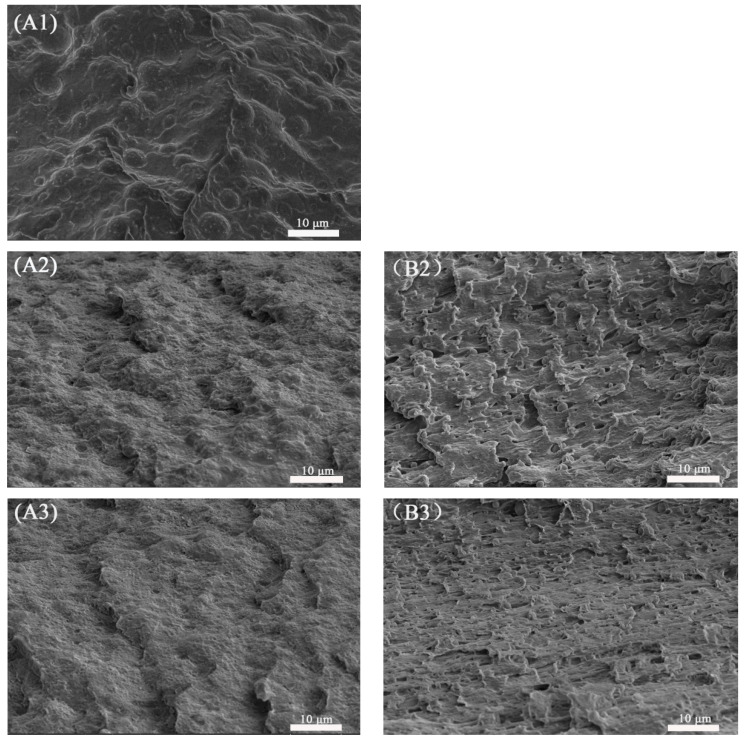

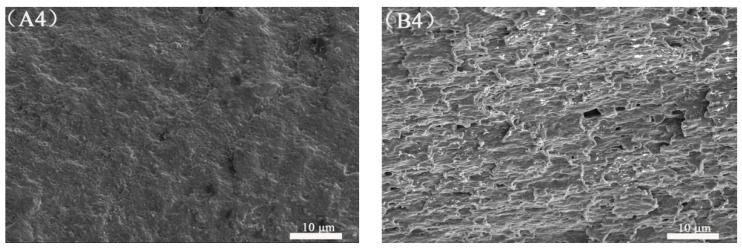
SEM images (×3000) of cryofractured surfaces of PETG/PGA/ADR tensile fractured specimens along tensile direction. (**A**) unstretched part ((**A1**)—65/35/0; (**A2**)—65/35/0.3; (**A3**)—65/35/0.6; (**A4**)—65/35/0.9); (**B**) stretching-oriented part ((**B2**)—65/35/0.3; (**B3**)—65/35/0.6; (**B4**)—65/35/0.9).

In order to research the micromorphological evolution of the PETG/PGA (65/35) samples with and without ADR during the tensile process, the cryofractured surfaces of the tensile fractured specimens along the tensile direction were photographed, including the stretching-oriented parts and the unstretched parts, as shown in Figure 13. The unstretched part of the PETG/PGA (65/35) sample (see Figure 13A) showed many oversized PGA particles (with diameters more than 5 μm) dispersed in the matrix. This could be attributed to the poor compatibility at the PETG/PGA interface and excessive amount of PGA. Owing to their brittle fracturing behavior (in terms of their low elongations at break), there was no obvious tensile yielding and necking observed in the PETG/PGA (65/35) specimens before they ruptured during the tensile testing process. Hence, SEM images of the part oriented in the tensile direction of the PETG/PGA (65/35) sample could not be included in Figure 13. In the case of the unstretched parts of the PETG/PGA/ADR (65/35/x) samples (see Figure 13(A2–A4), the PGA particles were seen embedded in the matrix with a blurred interface. Moreover, the domain size of the PGA decreased significantly upon the addition of ADR. The distribution of the diameters of the PGA particles and the apparent number-average particle sizes (${\overset{-}{d}}_{\mathrm{app}}$) of PETG/PGA (65/35) with different ADR contents were determined from the TEM images (see Figure 11 and Figure 12). The reactive compatibilization by ADR at the PETG/PGA interface was responsible for the decrease in the size of the PGA domains with a blurred interface, as discussed in the section “TEM observation”. 

Figure 13(B2–B4) show the SEM photographs of the stretching-oriented parts of the PETG/PGA/ADR (65/35/x) samples with different ADR contents. For the PETG/PGA/ADR (65/35/0.3) sample, as shown in Figure 13(B2), many PGA particles were seen debonded from the PETG matrix, accompanied by a highly oriented PETG matrix.

As compared to the oversized PGA particles of the PETG/PGA (65/35) binary sample, the finer PGA particles of the PETG/PGA/ADR (65/35/0.3) sample showed significant ability to induce the shear yielding of the surrounding matrix. The formation of massive shear bands was conducive to the termination of craze and a stable orientation. As a result, the tensile ductility of the ternary sample was significantly improved. With a further increase in the addition of ADR to 0.6 and 0.9 phr, the degree of the orientation of the PETG matrix increased, accompanied by the formation of a more intensively orientated structure. Moreover, for the parts oriented along the tensile direction, it is worth noting that the interfacial debonding of the PGA particles could be effectively restrained with an increase in ADR content. This demonstrates the efficient reactive compatibilization effect of ADR on the increase in the PETG/PGA interfacial adhesion. Overall, with an increase in ADR content, the domain size of the PGA decreased significantly. Additionally, the increased interfacial adhesion was more conducive to an increase in the tensile ductility of the PETG/PGA samples modified with ADR. However, compared with the oversized PGA particles of the PETG/PGA (65/35) binary sample, the finer PGA particles of the PETG/PGA/ADR (65/35/x) samples could result in more stress concentration around these particles under tensile loads, leading to a decrease in the inflexibility of the PETG matrix. As a result, the tensile moduli of the PETG/PGA/ADR (65/35/x) samples decreased slightly from 1509 to 1403 MPa when the ADR content increased from 0 to 0.9 phr, as shown in Table 4. 

In summary, the SEM results are consistent with the findings of the tensile testing results and TEM analysis. It was also confirmed that ADR had a pronounced reactive compatibilization effect, which tailored the interfacial adhesion and micromorphology of the PETG/PGA blends. As a result, a series of PETG/PGA/ADR blends with improved tensile performance were prepared.

#### 3.2.5. DSC and WAXD Measurements

**Figure 14:**
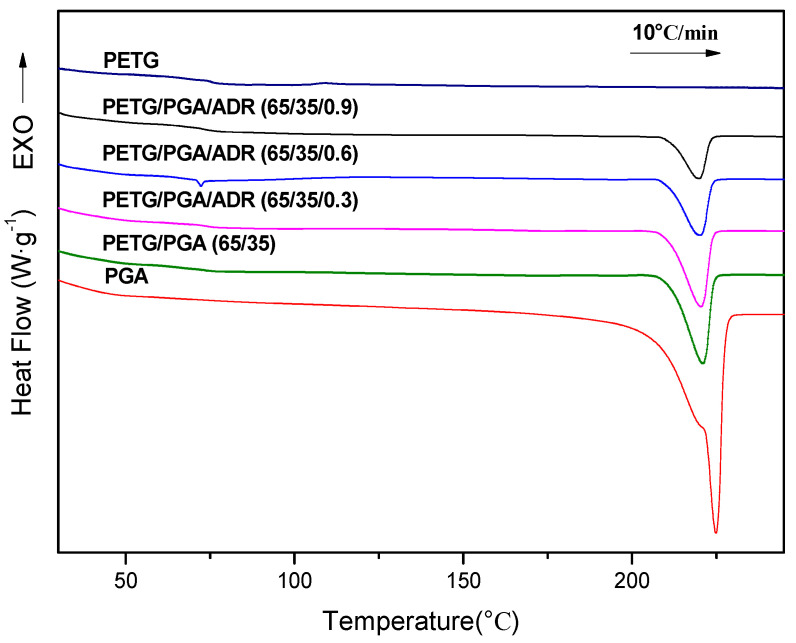
Heating DSC thermograms for neat PETG, PGA, and PETG/PGA/ADR (65/35/x) samples.

The effect of adding ADR on the crystallization properties of the PETG/PGA (65/35) samples with varying ADR contents was investigated by DSC and WAXD measurements.The heating DSC thermograms of the PETG/PGA/ADR samples are presented in Figure 14, and their corresponding thermodynamic parameters are summarized in Table 5. 

The DSC thermogram of PETG/PGA (65/35) showed a small step at 73.1 °C and a strong endothermic peak at 221.0 °C. They were attributed to the glass transition temperature of the amorphous PETG matrix and melting peak of the semicrystalline PGA component, respectively. It was confirmed by the data in Table 5 that there was no significant shift in the *T*g of the PETG matrix and *T*_m_ of the PGA component. However, the crystallinity of the PGA component in the PETG/PGA/ADR (65/35/x) samples decreased from 36.0 to 16.2% with an increase in ADR content from 0 to 0.9 phr. The increase in the irregularity of the PGA macromolecular segments caused by the chain extension/branching reactions between PGA, PETG, and ADR were responsible for the decrease in the crystallinity of PGA component. 

**Figure 15:**
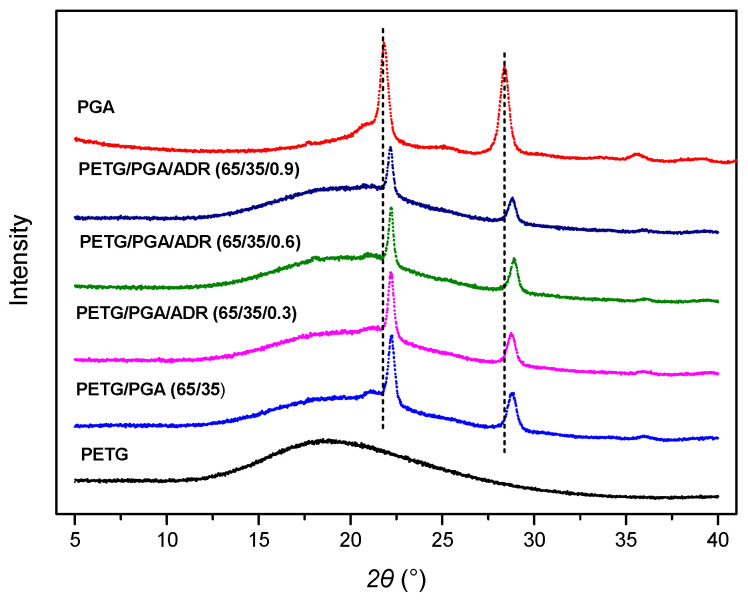
WAXD patterns of neat PETG, PGA, and PETG/PGA/ADR (65/35/x) samples.

The WAXD patterns of the PETG/PGA/ADR (65/35/x) samples are shown in Figure 15. Consistent with the DSC results, the WAXD patterns of neat PETG showed a dispersive diffraction peak in the range of 2θ = 10° to 35°, suggesting an amorphous nature for neat PETG. The PETG/PGA (65/35) sample showed two shaper peaks at 22.2° and 28.8°, corresponding to the (110) and (020) crystal planes of PGA crystals, respectively. Furthermore, the PETG/PGA (65/35) samples modified with ADR showed a gradual decrease in the peak intensities of the PGA component with an increase in ADR content. This could be attributed to the decrease in the crystallinity of the PGA component resulting from chain extension/branching reactions between PGA, PETG, and ADR. 

#### 3.2.6. Vicat Softening Temperature (VST)

The VST values of the neat PETG and PETG/PGA (65/35) samples modified with different ADR contents are listed in Table 6. The VST of neat PETG was only 81.0 °C. This could be attributed to the lower *T*_g_ (74.9 °C) of the amorphous PETG matrix. The three known ways to enhance the heat resistance of thermoplastic polymers include increasing the *T*_g_, increasing the crystallinity, and reinforcement with rigid inorganic/organic particles [31]. However, both increasing the *T*_g_ and increasing the crystallinity of the PETG matrix are inapplicable for the amorphous PETG resin, which cannot be crystallized. Hence, in this work, increasing the heat resistance of the PETG matrix mainly depended on the reinforcement effect of rigid PGA particles. As expected, the addition of 35 wt% PGA caused the VST of the PETG/PGA (65/35) sample to increase to 99.2 °C, which was 18.2°C higher than that of neat PETG. Based on the DSC results, the PGA particles dispersed in the PETG/PGA (65/35) sample had a semicrystalline nature with a higher *T*_m_ (221.0 °C) and crystallinity (36.0%). These rigid PGA particles having a higher *T*_m_ and crystallinity could prevent the deformation of the PETG matrix during heating. However, with an increase in the ADR concentration, the VST of the PETG/PGA/ADR (65/35/x) samples decreased gradually. For example, when the ADR content increased to 0.6 phr, the VST of the ternary sample decreased from 99.2 (PETG/PGA (65/35) binary sample) to 94.1 °C. As the ADR content increased to 0.9 phr, the VST further dropped to 90.5 °C. The weakening of reinforcing effect of PGA particles on the heat resistance of the PETG/PGA/ADR samples could be mainly attributed to the decreased crystallinity of the PGA particles. This was the result of chain extension/branching reactions between PGA, PETG, and ADR. 

## 4. Conclusions

In this paper, PGA was used as a reinforcing component to improve the mechanical properties of PETG. When the PGA content increased to 35 wt%, the tensile yield strength/tensile modulus of the PETG/PGA (65/35) sample increased from 44.38/1076 MPa for the neat PETG to 48.4/1509 MPa. Meanwhile, the VST increased from 81.0 to 99.2 °C. However, due to the poor interfacial compatibility between the PETG matrix and PGA domains, the tensile ductility of the PETG/PGA (65/35) sample decreased drastically. In addition, the high melt fluidity of PGA largely increased the MFR of the PETG/PGA blend, which was not conducive to its processing by some common processing technologies, such as extrusion, blown film, thermoforming, etc. 

In order to improve the interfacial compatibility and rheological properties of the PETG/PGA (65/35) blend, ADR was introduced as a reactive modifier. The torque and MFR testing showed that the melt viscosity of the PETG/PGA blend increased significantly with an increase in ADR content. In addition, micromorphological analysis indicated that ADR had a strong reactive compatibilizing effect on the blend, which increased the interfacial adhesion in the PETG/PGA, accompanied by a decrease in the PGA domain size. As a result, the tensile ductility of the PETG/PGA (65/35) blend modified with ADR was significantly improved, in terms of the elongation at break. DSC and WAXD analyses showed that the PETG matrix was amorphous and that the PGA component was semicrystalline in nature. Moreover, the crystallinity of the PGA decreased gradually with an increase in ADR content. The decrease in the crystallinity of the PGA component due to the addition of ADR reduced the reinforcing effect of PGA on the heat resistance of the PETG/PGA/ADR (65/35/x) samples. 

In summary, by carefully controlling the ADR concentration, the micromorphology, mechanical performance, and rheological properties of PETG/PGA blends modified with ADR can be effectively regulated and balanced for meeting the requirements of different applications in a better manner.

## Figures and Tables

**Table 1:** Compositions of PETG/PGA (y/z) and PETG/PGA/ADR (65/35/x) samples.

Sample Number	PETG (wt.)	PGA (wt.)	ADR (phr)
1#	100	0	0
2#	95	5	0
3#	85	15	0
4#	75	25	0
5#	65	35	0
6#	65	35	0.3
7#	65	35	0.6
8#	65	35	0.9

**Table 2:** Tensile performance of neat PETG and PETG/PGA (y/z) samples.

Samples	Tensile Yield Strength (MPa)	Tensile Modulus (MPa)	Elongation at Break (%)
Neat PETG	44.38 ± 0.62	1076 ± 35	223.0 ± 10.2
PETG/PGA (95/05)	45.26 ± 0.62	1145 ± 17	227.2 ± 8.9
PETG/PGA (85/15)	46.33 ± 0.20	1254 ± 07	260.4 ± 14.2
PETG/PGA (75/25)	47.57 ± 0.77	1370 ± 50	29.0 ± 6.5
PETG/PGA (65/35)	48.24 ± 0.72	1509 ± 31	11.1 ± 1.8

**Table 3:** Differential scanning calorimetry (DSC) parameters of neat PGA, PETG, and PETG/PGA (y/z) samples.

Samples	*T*_g_ (°C) (PETG)	*T*_m_ (°C) (PGA)	Δ*H*_m_ (J/g) (PGA)	*X*_c_ (%) (PGA)
Neat PGA	N/A	224.9	−74.67	37.7
Neat PETG	74.9	N/A	N/A	N/A
PETG/PGA (95/05)	74.7	221.2	−2.91	29.4
PETG/PGA (85/15)	74.1	221.6	−9.48	31.9
PETG/PGA (75/25)	72.3	221.1	−17.49	35.3
PETG/PGA (65/35)	73.1	221.0	−24.96	36.0

**Table 4:** Tensile performance of neat PETG and PETG/PGA/ADR (65/35/x) samples.

Samples	Tensile Yield Strength (MPa)	Tensile Modulus (MPa)	Elongation at Break (%)
Neat PETG	44.38 ± 0.62	1076 ± 35	223.0 ± 10.2
PETG/PGA (65/35)	48.24 ± 0.72	1509 ± 31	11.1 ± 1.8
PETG/PGA/ADR (65/35/0.3)	48.73 ± 0.69	1512 ± 15	123.2 ± 11.4
PETG/PGA/ADR (65/35/0.6)	48.06 ± 0.80	1450 ± 20	135.4 ± 5.1
PETG/PGA/ADR (65/35/0.9)	48.43 ± 1.28	1403 ± 58	160.8 ± 9.4

**Table 5:** DSC parameters of neat PGA, PETG, and PETG/PGA/ADR (65/35/x) samples.

Samples	*T*_g_ (°C) (PETG)	*T*_m_ (°C) (PGA)	Δ*H*_m_ (J/g) (PGA)	*X*_c_ (%) (PGA)
Neat PGA	N/A	224.9	−74.67	37.7
Neat PETG	74.9	N/A	N/A	N/A
PETG/PGA (65/35)	73.1	221.0	−24.96	36.0
PETG/PGA/ADR (65/35/0.3)	73.3	220.4	−22.15	32.0
PETG/PGA/ADR (65/35/0.6)	71.9	220.1	−16.25	23.6
PETG/PGA/ADR (65/35/0.9)	72.6	219.9	−11.28	16.4

**Table 6:** VSTs of neat PETG and PETG/PGA/ADR (65/35/x) samples.

Samples	VST (°C)
Neat PETG	81.0
PETG/PGA (65/35)	99.2
PETG/PGA/ADR (65/35/0.3)	95.0
PETG/PGA/ADR (65/35/0.6)	94.1
PETG/PGA/ADR (65/35/0.9)	90.5

## Data Availability

The data presented in this study are available on request from the corresponding author and the first author.
